# Antimicrobial prescribing in dogs and cats with urinary tract disease in a prospective intervention trial

**DOI:** 10.1093/jvimsj/aalaf054

**Published:** 2026-01-21

**Authors:** Amy W Hii, James R Gilkerson, Kirsten E Bailey, Ri Scarborough, Anna E Sri, Brian Hur, Magdoline Awad, Lynsey Etheridge, Laura Y Hardefeldt

**Affiliations:** Asia-Pacific Centre for Animal Health, Melbourne Veterinary School, Faculty of Science, University of Melbourne, Parkville, Victoria, Australia; National Centre for Antimicrobial Stewardship, Peter Doherty Institute, Carlton, Victoria, Australia; Asia-Pacific Centre for Animal Health, Melbourne Veterinary School, Faculty of Science, University of Melbourne, Parkville, Victoria, Australia; National Centre for Antimicrobial Stewardship, Peter Doherty Institute, Carlton, Victoria, Australia; Asia-Pacific Centre for Animal Health, Melbourne Veterinary School, Faculty of Science, University of Melbourne, Parkville, Victoria, Australia; National Centre for Antimicrobial Stewardship, Peter Doherty Institute, Carlton, Victoria, Australia; Asia-Pacific Centre for Animal Health, Melbourne Veterinary School, Faculty of Science, University of Melbourne, Parkville, Victoria, Australia; National Centre for Antimicrobial Stewardship, Peter Doherty Institute, Carlton, Victoria, Australia; Asia-Pacific Centre for Animal Health, Melbourne Veterinary School, Faculty of Science, University of Melbourne, Parkville, Victoria, Australia; National Centre for Antimicrobial Stewardship, Peter Doherty Institute, Carlton, Victoria, Australia; Asia-Pacific Centre for Animal Health, Melbourne Veterinary School, Faculty of Science, University of Melbourne, Parkville, Victoria, Australia; National Centre for Antimicrobial Stewardship, Peter Doherty Institute, Carlton, Victoria, Australia; Greencross Pet Wellness Company, North Ryde, New South Wales, Australia; Greencross Pet Wellness Company, North Ryde, New South Wales, Australia; Asia-Pacific Centre for Animal Health, Melbourne Veterinary School, Faculty of Science, University of Melbourne, Parkville, Victoria, Australia; National Centre for Antimicrobial Stewardship, Peter Doherty Institute, Carlton, Victoria, Australia

**Keywords:** antimicrobials, bacteria, culture and susceptibility testing, cystitis, stewardship, urinalysis

## Abstract

**Background:**

Veterinarians often cite the cost as a barrier to performing culture and susceptibility (C&S) testing.

**Hypothesis/Objectives:**

To investigate the effects of fee-free C&S and to a decision support tool for urinary tract disease on antimicrobial prescribing behavior.

**Animals:**

Twenty-one small animal general practice veterinary clinics in Melbourne, Australia were included in the study, 10 clinics in the intervention group and 11 clinics in the control group. Urine samples collected from dogs and cats were eligible.

**Methods:**

A prospective cohort study was conducted in which clinics in the intervention group were provided access to free urine C&S for dogs and cats with clinical signs of urinary tract disease and a decision support tool. Clinical histories were analyzed for both groups to determine antimicrobial prescribing behavior.

**Results:**

A total of 480 urine C&S submissions were received from the intervention group and 636 consultations for patients with urinary tract disease were included in the control group. Patients in the control group were more likely to empirically prescribe antimicrobials (*P* = .04). A higher proportion of cats in the control group received cefovecin (48%) compared with cats in the intervention group (22%, *P* = .03). Patients in the intervention group also were more likely to be prescribed empirical antimicrobial treatment for a shorter period of time than in the control group (*P* ≤ .001).

**Conclusions and clinical importance:**

Removing the cost barrier to microbiological diagnostic testing and the provision of a decision support tool resulted in positive changes to antimicrobial prescribing behavior, particularly decreased prescribing frequency and shorter duration of antimicrobial treatment.

## Introduction

Antimicrobial resistance is a public health concern, and antimicrobial use is contributing to the problem. Antimicrobial stewardship (AMS) efforts are targeted at promoting appropriate and responsible use of antimicrobials to ensure effective treatments remain available for future generations.^1^^,^^2^ Bacterial cystitis is a common reason for prescribing antimicrobials in companion animal general practice in Australia.^3^^,^^4^ Culture and susceptibility (C&S) testing is recommended for all patients with clinical signs of urinary tract disease (ie, stranguria, hematuria, polyuria, dysuria, or a combination of these signs) to ensure that an effective antimicrobial is used.^5^^,^^6^

Previous qualitative research has identified several barriers to conducting microbiological diagnostic testing, including the owner’s unwillingness to pay for laboratory testing, challenges in collecting samples, and delays in treatment while awaiting results.^6–8^ In most countries, including Australia, all veterinary expenses are the responsibility of the pet owner. Hence, the client’s financial resources often come into consideration when deciding whether diagnostic testing such as C&S is performed.^8^ Various AMS interventions such as educational webinars, prescribing guidelines, delayed prescribing, and drug auditing have been assessed for their effects on antimicrobial use,^9^ but currently, no intervention studies that investigate the impact of the cost of microbiological diagnostic testing combined with decision support are available.

We aimed to investigate how removing the cost of C&S and implementing a decision support tool would influence the prescribing behavior of veterinarians. The clinical decisions made after receiving C&S results also were examined.

## Materials and methods

The intervention study has been described previously.^10^ Briefly, the intervention group consisted of 10 general practice veterinary clinics from a large corporate group in metropolitan Melbourne, Australia that voluntarily enrolled in the study. The clinics in the intervention group were provided free C&S testing for urine samples from dogs and cats (4th January to 3rd December 2022). A decision support tool was provided for guidance on empirical treatment recommendations for sporadic bacterial cystitis based on local prescribing guidelines.

The control group consisted of 11 general practice veterinary clinics distributed across the same geographical area of metropolitan Melbourne, all belonging to the same corporate group. These practices did not receive free C&S or the decision support tool. In the control group, all patients that had urinalysis (UA) performed were included. Table 1 summarizes the characteristics of the clinics in the intervention and control groups.

**Table 1:** Details of clinics in each study group.

	**Intervention**	**Control**
**Number of clinics**	10	11
**Location**	Metropolitan Melbourne, Victoria	Metropolitan Melbourne, Victoria
**Study period**	January to December 2022	January to December 2022
**Socioeconomic**	All in advantaged areas	All in advantaged areas
**Full-time equivalent (FTE) veterinarians**	25	22.5
**Number of consultations in 2022**	30 249	39 559

The results of the C&S were reported to the clinics by a commercial laboratory using its standardized protocols including guidance on thresholds for significance based on (colony-forming units)/mL according to sample collection method.

Clinical histories for all patients in the study were obtained from VetCompass Australia.^11^ Full patient histories were available for the study cohort but only records spanning 3 months before and 6 weeks after the UA were obtained for the control group. Records from intervention and control cases were manually examined by 1 of 3 authors (A.H., R.S., and L.H.). Data on treatment, clinical signs, results of in-house cytology, and action taken after receipt of C&S results were recorded (described below).

### Adjunctive treatment

Adjunctive treatments were defined as medications prescribed during presentation for the purposes of analgesia or anxiolysis in relation to the patient’s urinary clinical signs. Treatments were categorized into one of the following groups: nonsteroidal anti-inflammatory drugs (NSAIDs), opioids, gabapentin, anxiolytics, and incontinence medications. Gabapentin was analyzed separately because it is used for its anxiolytic and analgesic properties in cats.^12^

Other adjunctive treatments prescribed included incontinence medications (phenylpropanolamine and stilbesterol or estriol). Nonprescription treatments recommended for the purpose of minimizing anxiety such as feline pheromones (Feliway; Ceva), milk proteins (Zylkene; Vetoquinol), and urinary support supplements (Cystopro and Cystophan; ADM Protexin Vet) also were noted.

### Clinical signs

Urinary clinical signs were classified into the following categories: hematuria, stranguria, polydipsia without polyuria, polyuria without polydipsia, polyuria and polydipsia, urinary incontinence or house soiling, and genital abnormalities. Urinary incontinence was grouped with house soiling because of difficulty distinguishing between the 2 if the owner did not observe the urination. Genital abnormalities included any external abnormalities involving the vulva, prepuce, penis, or peri-genital areas such as skin lesions, discharge, and licking.

If the patient had no urinary signs and the urine sample was submitted for C&S for other reasons, such as part of the diagnostic evaluation for another condition, this was classified as “no urinary clinical signs.” Samples submitted for reassessment after the initial presentation were classified as “follow-up.”

### Subclinical bacteriuria

Subclinical bacteriuria was defined as cases where the urine sample was positive for bacterial growth from a patient without urinary clinical signs.^5^ Detailed examination of subclinical bacteriuria cases was undertaken only in the intervention cohort.

### In-house urinalysis and cytology

Data were collected on results of in-house UA and cytology (presence of red blood cells, white blood cells, bacteria, urinary crystals, or some combination of these). Some clinics performed cytology using Diff-quik staining, whereas some had Idexx Sedivue (a veterinary urine sediment analyzer) available. These results were compared to reports from the diagnostic laboratory to evaluate consistency with culture results. If the clinical history did not mention the presence of bacteria (rods or cocci), it was assumed that the clinician did not observe any.

### Empirical antimicrobials

Empirical antimicrobial treatment in the study refers to any antimicrobials prescribed or administered before C&S results were received. To evaluate the empirical use of antimicrobials, only the initial consultation submission where patients were reported to have urinary clinical signs was considered, and all follow-up submissions were excluded. If antimicrobials were empirically prescribed, the agent and duration of treatment were recorded. The duration of treatment for cefovecin, a long-acting injectable third-generation cephalosporin was considered to be 14 days. It was assumed that the owner completed the course of antimicrobials as dispensed, if not instructed otherwise. Patient weight was not consistently recorded and thus appropriateness of dosing could not be evaluated.

### Post-C&S actions

Alterations to antimicrobial treatment after receiving C&S results were assessed to determine if recommendations were given to finish (complete) the course, discontinue, de-escalate, escalate, or change antimicrobials. Follow-up submissions were excluded. In instances where the patient was given cefovecin, it was considered as “finished course” because it was not possible to change, discontinue, or de-escalate this antimicrobial. The cases were classified as “no action” if no antimicrobials were prescribed before or after receiving C&S results. This approach may have included situations where antimicrobials were not prescribed, and the clinician proceeded to undertake further investigations.

The variables were selected based on clinically relevant comparisons. Variables compared included frequency of adjunctive treatment, clinical signs (hematuria, stranguria, polydipsia, polyuria, house soiling, and discharge or licking or abnormality of external genitalia), cytology findings (bacteria, crystals, red blood cells, white blood cells, and concordance with C&S results), and empirical treatment and post-C&S actions. Clinical signs and cytology findings were obtained from clinical notes. Empirical and adjunctive treatment were recorded from the items charged and medication label. The post-C&S actions were recorded from the clinical notes, items charged, and medication label. All data were recorded without transformation or reclassification. Cat and dog results were compared collectively (intervention vs control) as well as separately.

The data were compiled and recorded in Filemaker Pro and Microsoft Excel. Statistical analyses were performed using Microsoft Excel and R studio (September 1, 2023). Test of proportions for comparing 2 groups and Mann–Whitney *U* tests were used when data were not normally distributed.

## Results

The intervention group consisted of 366 unique patients associated with 480 urine C&S submissions. Most submissions were from dogs (296/480; 62%) and the remainder from cats (184/480; 38%). During the study period, 285 patients had 1 C&S, 56 patients had 2 C&S, 17 patients had 3 C&S, 3 patients had 4 C&S, and 4 patients had 5 C&S.

Of the cases where the patient was presented with urinary clinical signs, 237 unique patients presented with 253 cases of sporadic cystitis, with the remaining 20 unique patients presenting with 44 cases of recurrent cystitis. There were 29 cases of subclinical bacteriuria from 28 unique patients and 38 cases of patients with no clinical signs for which urine culture was negative.

In the control group, clinical records for 636 consultations that included UA from 444 unique patients were reviewed, with 68% from dogs (433/636) and the remainder from cats (203/636; 32%). Urine samples were submitted for C&S in 29 consultations in the control group (4.6%) of which 20 were from dogs (4.6%) and 9 from cats (4.4%). No difference in the rate of C&S submissions was found between dogs and cats in the control group (*P* = .9).

### Adjunctive treatment

Adjunctive treatments (of any type) were prescribed to 16% (51/296) of dogs and 48% (89/184) of cats in the intervention group and to 17% (72/433) of dogs and 49% (99/208) of cats in the control group. Analgesia prescribed included NSAIDs (meloxicam, robenacoxib, carprofen, grapiprant, and piroxicam), opioids (buprenorphine, methadone, tramadol, and codeine), and paracetamol, with NSAIDs being the most commonly dispensed adjunctive treatment (Table 2).

**Table 2:** Adjunctive therapy prescribed for patients presented for urinary complaints.

**Medication**		**Intervention**		**Control**	
		**Dogs** ***n* = 296** ***N* (%)**	**Cats** ***n* = 184** ***N* (%)**	**Dogs** ***n* = 433** ***N* (%)**	**Cats** ***n* = 204** ***N* (%)**
**Analgesia**	NSAIDs	40 (14)	32 (17)	59 (14)	65 (32)
	Opioids	13 (4.4)	41 (22)	4 (0.9)	38 (19)
	Paracetamol	4 (1.4)	0 (0)	0 (0)	0 (0)
	Both NSAIDs and opioids	3 (1.0)	9 (4.9)	0 (0.0)	14 (6.9)
	Total receiving analgesia	48 (16)	69 (38)	63 (15)	88 (43)
**Gabapentin**		3 (1.0)	23 (12.5)	11 (2.5)	17 (8.4)
**Anxiolytic**	Fluoxetine	2 (0.7)	2 (1.1)	1 (0.2)	1 (0.5)
	Clomipramine	0 (0.0)	4 (2.2)	0 (0.0)	4 (2.0)
	Zylkene	1 (0.3)	9 (4.9)	1 (0.2)	8 (3.9)
	Total receiving anxiolytics	3 (1.0)	15 (8.2)	2 (0.5)	13 (6.4)

Abbreviation: NSAIDs = nonsteroidal anti-inflammatory drugs.

Cats in both the intervention (*P* ≤ .001) and control (*P* ≤ .001) groups were more likely to be prescribed analgesia (NSAIDs, opioid, or a combination) compared with dogs. Gabapentin was prescribed for 5.4% (26/480) of cases in the intervention study and 4.5% (28/626) of cases in the control group (*P* = .47). Cats were also more likely than dogs to be dispensed gabapentin in both intervention (*P* ≤ .001) and control (*P* ≤ .001) groups.

### Clinical signs

No difference was found between the prevalence of clinical signs in the intervention (297/480; 62%) and control groups (381/636; 60%; *P* = .5). The most common presenting complaints were house soiling or urinary incontinence, followed by hematuria and stranguria (Table 3).

**Table 3:** Presenting complaints for patients in intervention and control groups.

**Clinical signs**	**Intervention** ***n* = 480** ***N* (%)**	**Control** ***n* = 636** ***N* (%)**
**No urinary clinical signs**	109 (23)	133 (21)
**House soiling/urinary incontinence**	95 (20)	156 (25)
**Hematuria**	86 (18)	98 (15)
**Stranguria**	71 (15)	86 (14)
**Polyuria without polydipsia**	66 (14)	67 (11)
**Follow-up**	56 (12)	89 (14)
**Polydipsia and polyuria**	55 (11)	53 (8.3)
**Discharge, licking, or abnormality of external genitals**	31 (6.5)	61 (9.6)
**Polydipsia without polyuria**	20 (4.2)	36 (5.7)
**No notes/Unknown**	18 (3.8)	29 (4.6)

Some of the common reasons for performing C&S for patients that were not presented with urinary clinical signs included monitoring chronic comorbidities such as chronic renal disease, hyperthyroidism, and diabetes mellitus, diagnostic evaluation for nonspecific signs such as inappetence and pyrexia, and further investigation of proteinuria and microscopic hematuria.

### Subclinical bacteriuria

Results from most C&S submissions in the control group (15/29; 52%) were not available and thus outcomes could not be assessed.

Subclinical bacteriuria was identified from the clinical records in 6% (29/480) of C&S submissions in the intervention group. No significant difference was found in occurrence between dogs (19/296; 6.4%) and cats (10/184; 5.4%; *P* = .67), or between voided and cystocentesis samples (*P* = .56). Antimicrobials were prescribed empirically in 41% of subclinical bacteriuria cases (12/29), with amoxycillin-clavulanic acid most frequently prescribed (7/12; 58%). Duration of treatment most commonly was 7 days (6/12; 50%) followed by 14 days (3/12; 25%). *Escherichia coli* was the most commonly isolated bacterium from subclinical bacteriuria samples (20/29; 69%) and 45% of samples had pure growth of *E coli* (Supplementary Material S1).

**Figure 1:**
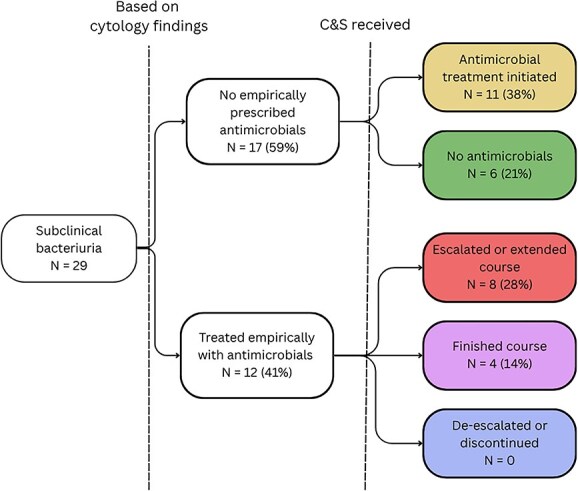
Outcomes from cases of subclinical bacteriuria from the intervention group.

Antimicrobial treatment was initiated after C&S results became available in 65% (11/17) of cases. When an antimicrobial had already been prescribed empirically, 8/12 (67%) cases had the antimicrobials escalated to a higher importance rated antimicrobial, the course was extended, or a second antimicrobial was added after C&S results. In 2 cases, animals that had received cefovecin empirically in the previous 14 days additionally were started on fluoroquinolones. No instances of de-escalation or discontinuation of empirically used antimicrobials were noted (Figure 1).

### Cytology

In-house UA was performed in 64% (306/473) of cases in the intervention group. Urine specific gravity, urine dipstick testing, stained sediment examination, and unstained sediment examination typically were performed. However, in many records, it was not possible to determine what type of sediment examination was performed.

In 7 cases, it was not clear if UA was performed, and these cases were excluded. Where UA was not performed, 37/167 (22%) were follow-up samples. On stained sediment examination, red blood cells were the most frequently detected abnormality (121/306; 40%), followed by bacteria (118/306; 39%; Table 4). No difference was found in the prevalence of each cytological finding between cystocentesis and voided samples, and the accuracy for both was similar when compared to C&S findings.

Most cytology (198/306; 65%) matched C&S results for detection of bacteria and type of bacteria detected. However, included were 7 cases where the clinician did not detect all of the bacterial types present in the C&S culture and 6 cases where both bacterial morphologies were noted when only 1 type was identified by C&S.

Of the 89 cases (89/306; 29%) where the cytology findings did not match the C&S results, the most common difference was detecting bacteria when culture results were “negative” or “not significant” (51/89; 57%), followed by failing to detect any bacteria on cytology when the culture was positive for bacterial growth (32/89; 36%). There were 6 cases (6.7%) where the clinician detected a morphology of bacteria that was not detected on culture.

The remaining 6% of cases did not have sufficient details of the UA results recorded to determine if the cytology findings matched the C&S results.

### Empirical treatment

“Follow-up” cases were excluded for the purposes of analyzing antimicrobials prescribed empirically when urine investigations were conducted. Patients in the control group (179/378; 47%) were more likely to be prescribed antimicrobials empirically than those in the intervention group (111/296; 38%; *P* = .01). Within the intervention group, dogs were more likely than cats to receive antimicrobials empirically (*P* = .01) but no difference was found in empirical prescribing between dogs and cats in the control group (Table 5). The proportion of patients prescribed antimicrobials empirically based on the presence of urinary clinical signs and presence of bacteria seen on cytology is presented in Supplementary Material SS2.

**Table 4:** In-house urinalysis cytology findings from intervention cases, performed either using stained dry mount technique or Sedivue.

**Cytology findings**	**Number of cases** ***n* = 306** ***N* (%)**
**Red blood cells**	121 (40)
**Leukocytes**	92 (30)
**Bacteria** ** Rods/bacilli** ** Cocci** ** Unknown morphology**	87 (29) 52 (17) 15 (5.0)
**No significant findings**	70 (23)
**Crystals (all types)**	34 (11)
**Unknown/no notes**	19 (6.2)

**Table 5:** Proportion of patients with urinary clinical signs prescribed empirical antimicrobials.

	**Intervention**		**Control**	
	**Dogs (%)**	**Cats (%)**	**Dogs (%)**	**Cats (%)**
**No**	103 (55)	73 (67)	129 (50)	65 (55)
**Yes**	79 (42)	32 (29)	127 (49)	52 (44)
**Already on antimicrobials**	5 (2.7)	4 (3.7)	3 (1.2)	2 (1.7)
**Total**	187	109	259	119

Amoxicillin-clavulanate was the most frequent antimicrobial prescribed empirically in both dogs and cats, followed by cefovecin in cats, where a higher proportion in the control group (23/52; 44%) received cefovecin compared with the intervention group (7/32; 22%; *P* = .04; Table 6). The use of the prescribing guideline-recommended amoxicillin and trimethoprim-sulfamethoxazole (TMS) was low, 0%-4.6% and 0%-3%, respectively.

**Table 6:** Empirical antimicrobials prescribed in urinary tract cases, antimicrobials prescribed to less than 2 cases were excluded.

**Antimicrobials**	**Intervention**		**Control**	
	**Dogs** ***n* = 79** ***N* (%)**	**Cats** ***n* = 32** ***N* (%)**	**Dogs** ***n* = 127** ***N* (%)**	**Cats** ***n* = 52** ***N* (%)**
**Amoxicillin-clavulanate**	62 (78)	22 (69)	106 (83)	22 (42)
**Cefovecin**	0 (0)	7 (22)	1 (0.8)	23 (44)
**Cephalexin**	6 (7.6)	1 (3.1)	8 (6.3)	0 (0)
**Amoxicillin**	4 (5.1)	0 (0)	7 (5.5)	2 (3.8)
**Trimethoprim- sulfamethoxazole**	3 (3.8)	0 (0)	0 (0)	0 (0)
**Enrofloxacin**	2 (2.5)	0 (0)	4 (3.1)	2 (3.8)
**Marbofloxacin**	2 (2.5)	0 (0)	0 (0)	0 (0)
**Doxycycline**	0 (0)	0 (0)	1 (0.8)	2 (3.8)

**Figure 2:**
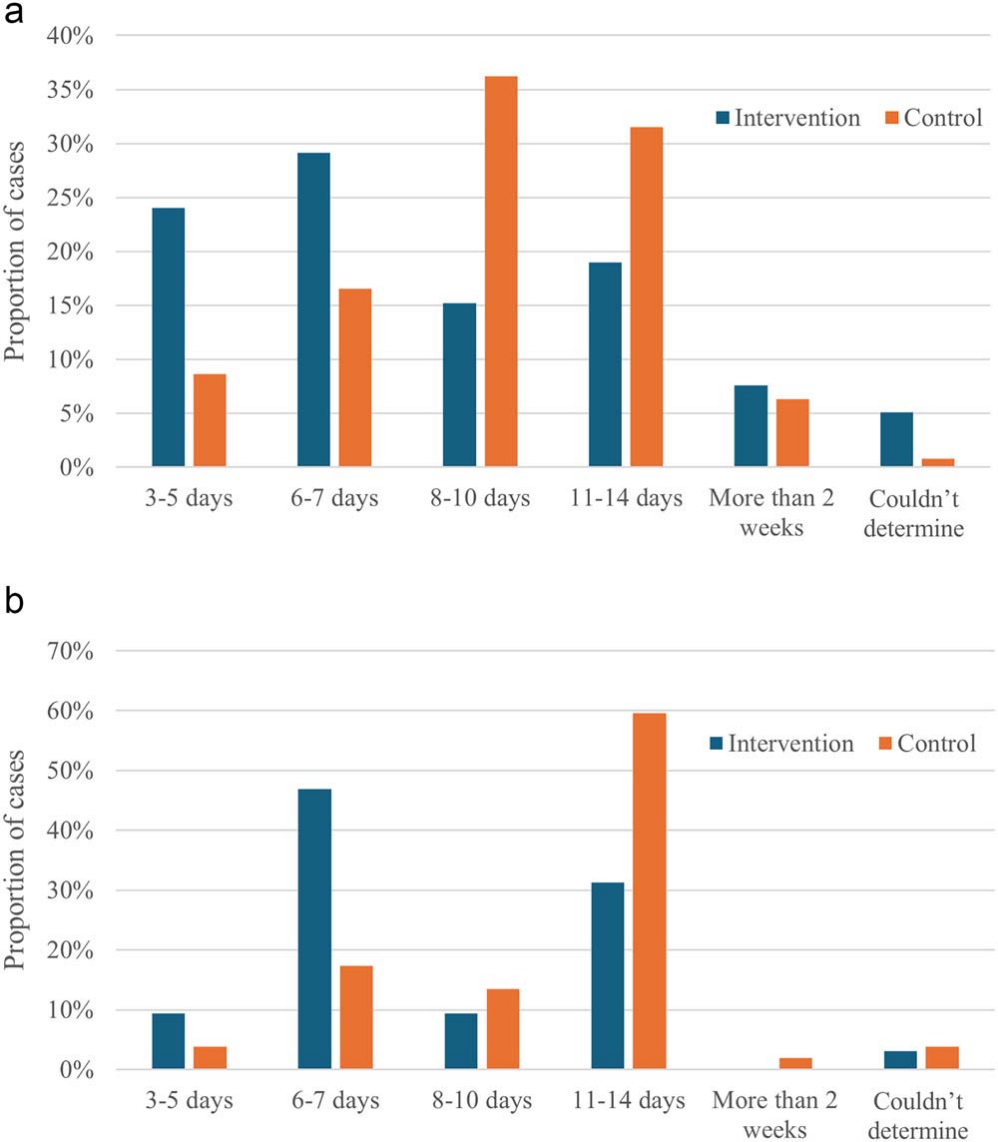
(a) Comparison of duration of empirical treatment with antimicrobials prescribed between dogs in the intervention and control groups. (b) Comparison of duration of empirical treatment with antimicrobials prescribed between cats in the intervention and control groups.

The intervention group had a higher proportion of cases with shorter durations of empirical antimicrobial treatment of 7 days or fewer compared with the control group (Figure 2a and b). However, nearly a quarter of dogs in the intervention group still were prescribed treatment for 11-14 days. The median duration of empirical antimicrobial treatment prescribed in the intervention study was 7 days compared with 10 days in the control group, which was 10 days (*P* ≤ .001).

No difference was found in type of antimicrobials prescribed for empirical treatment or for nonempirical use (Supplementary Material SS3).

### Post-C&S action

**Figure 3:**
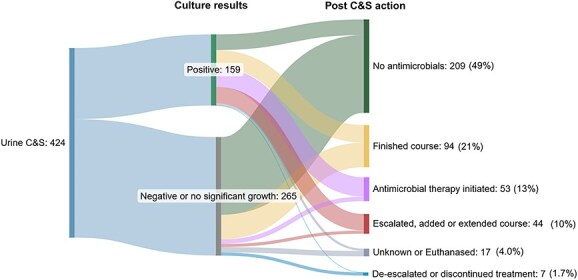
Outcomes after receiving C&S results from the intervention group. Abbreviation: C&S = culture and susceptibility.

There were 265 C&S tests that returned with results that were “negative” or “no significant growth” (63%) once follow-up cultures were excluded. A large proportion (185/265, 70%) did not have antimicrobials prescribed empirically. Of the cases where empirical treatment was initiated (140/424; 33%), there were only 7 instances (7/140; 5.0%) in which the clinician instructed the owner to discontinue the antimicrobial prescribed empirically (Table 7; Figure 3).

**Table 7:** Decisions around antimicrobial prescribing in the intervention group after C&S results received.

**Post C&S action**	**Overall (%)**	**Dogs (%)**	**Cats (%)**
**No action (no antimicrobials before or after)**	209 (49)	119 (46)	90 (55)
**Continue/finish empirical antimicrobials**	94 (22)	64 (25)	30 (18)
**Start antimicrobials based on C&S**	53 (13)	30 (11)	23 (14)
**Extend empirical antimicrobials**	30 (7.1)	24 (9.2)	6 (3.7)
**Unknown/not written in history**	14 (3.3)	6 (2.3)	8 (4.9)
**Escalate antimicrobials**	10 (2.4)	7 (2.7)	3 (1.8)
**Discontinue antimicrobials**	7 (1.7)	5 (1.9)	2 (1.2)
**Euthanized before C&S results back**	3 (0.7)	3 (1.1)	0 (0)
**Added fluroquinolones to cefovecin**	3 (0.7)	2 (0.8)	1 (0.6)
**Change antimicrobials (same importance rating)**	1 (0.2)	1 (0.4)	0 (0)
	**424**	261	163

Abbreviation: C&S = culture and susceptibility.

Of the cases where antimicrobial treatment was escalated after C&S results were received (10/140; 7.1%), 6 of the culture results showed susceptibility to a low importance antimicrobial such as amoxicillin or TMS and 2 cultures were negative for growth.

Most clinical records in the control group where urine was submitted for C&S (15/29; 52%) contained insufficient information on the organism and the susceptibility results to assess prescribing decisions post-C&S.

### Previous urinary concerns

Patient histories were assessed for the presence of any previous urinary concerns where the patient presented with one or more urinary clinical signs. Full patient histories were only available for the intervention group, and thus cases from the control group were excluded. It was determined that 135 unique patients had previously reported urinary concerns. No significant difference was found between the proportion of C&S submitted from dogs (103/217; 47%) and cats (66/149, 44%) with history of urinary disease (*P* = .55).

The frequency of empirical antimicrobial treatment was not different between cases with (39/102; 38%) or without (52/156; 33%) previous urinary concerns (*P* = .42). A significant difference was found between the duration of antimicrobial treatment for the group without previous urinary concerns (median, 7 days; mean, 8 days) and those with a history of urinary disease (median, 8 days; mean, 9.5 days; *P* = .03) for the treatment of sporadic cystitis.

### Follow-up cultures

There were 56 (12%) urine samples submitted for C&S as a follow-up sample after the initial presentation in the intervention group. Outcomes could not be evaluated in the control group because insufficient details of the follow-up conversations were rarely recorded.

In the intervention study, most (49/56, 88%) follow-up cultures were performed 1-2 weeks after the initial presentation and first C&S, including 7 instances (14%) where the earlier C&S test returned as “negative” or “no significant growth.” Post-C&S actions were not different between the “follow-up” and initial case groups. In half of the cases (29/56; 52%), antimicrobials were not prescribed before or after C&S results were received. The next most common decision was to complete the course of empirical treatment prescribed (10/56; 18%).

### Concordance with treatment guidelines

After performing cytology during in-house UA, veterinarians managing 174/306 (57%) of cases followed treatment guide recommendations^5^^,^^13^ displayed on the decision support tool.^10^ When bacteria of any morphology were seen (136/306; 44%), many veterinarians chose to delay prescribing antimicrobials (53/136; 39%) but there were only 2 instances (2/136; 1.5%) where a low importance antimicrobial (both amoxicillin) was used as recommended. If bacteria were not detected on cytology (170/306, 56%), 71% (121/170) of cases did not have antimicrobial treatment empirically prescribed, which is concordant with treatment guideline recommendations.^5^^,^^13^

After C&S results were received, antimicrobials were not prescribed for 64% (197/310) of the cases with “negative” or “no significant growth” results but empirical antimicrobial treatment was discontinued in only 2.6% (7/310) of cases.

## Discussion

Opportunities were identified to improve urine C&S use consistent with treatment guideline adherence for the treatment of bacterial cystitis. The interventions in our study promoted delayed prescribing, decreased the duration of empirical antimicrobial treatment and encouraged discontinuation or de-escalation of antimicrobial treatment. However, overall, a large proportion of treatment durations were still longer than current guideline recommendations (3-5 days) and veterinarians often performed follow-up C&S at the end of antimicrobial treatment, which is not recommended in prescribing guidelines.^5^^,^^13^

### Empirical use of antimicrobials

A shift toward a shorter duration of empirical treatment was observed in the intervention group (7 days) as compared with the control group (10 days), but both were longer than the guideline-recommended treatment duration of 3-5 days for sporadic bacterial cystitis and reinfections.^5^^,^^13^ Differentiating between the types of recurrent bacterial cystitis based on the clinical histories was not possible, and thus the appropriateness of a longer duration of treatment could not be assessed for the patients with previous urinary concerns.

Efforts should be made to identify comorbidities and risk factors in cases of recurrent cystitis because treatment success often is dependent on how well these factors are managed.^5^^,^^13^ Cases with poor response to initial antimicrobial treatment also should be reviewed for treatment compliance, dosing and frequency, as well as evaluated for any underlying conditions that may not have been initially apparent.

In the control group, where most cases (95%) did not have C&S, no alterations were made to the duration of the course of antimicrobials initially dispensed. Antimicrobial treatment was not de-escalated or discontinued, likely because the veterinarians in our study did not review the patient until the end of the course or a few days after. Any changes to the treatment plan, such as referral to a specialist, escalation of antimicrobials, or collection of samples for C&S after treatment failure were performed after completion of the antimicrobial course.

In contrast, when C&S was performed, an opportunity was created for the veterinarian to assess the pet’s case and response to antimicrobial treatment when conveying the results of laboratory tests. However, with the empirical use of cefovecin (contrary to the product information and guidelines), such opportunities to de-escalate or discontinue antimicrobial treatment are removed.^5^^,^^13^^,^^14^ In veterinary general practice, sporadic cystitis is commonly treated empirically, but improvements to antimicrobial prescribing can be made by following treatment guidelines.

### Adjunctive treatment

Cats were treated with analgesics and gabapentin more commonly than dogs. This difference is most likely because feline idiopathic cystitis is one of the most common differential diagnoses for urinary clinical signs in cats.^15^^,^^16^ Acute pain and anxiety or stress are prominent components of this disease, therefore clinicians may be more mindful of providing analgesia either in the form of NSAIDs or opioids, and gabapentin for its anxiolytic properties.^15–17^ However, anxiolytic-specific drugs such as fluoxetine and clomipramine were not frequently prescribed. Calming supplements or aids such as Zylkene (Vetoquinol) and Feliway (Ceva) may not be consistently recorded because these are over-the-counter products that can be purchased at the clinic without a digital record, or at pet stores.

Gabapentin often is prescribed for analgesia by general practice veterinarians.^18^ In companion animals, limited evidence is available that gabapentin is effective for acute pain, and its use for such reason is extra-label. Gabapentin should be used in conjunction with other forms of analgesia, particularly over the short term.^12^^,^^18^ Analysis of the clinical records for the precise rationale for gabapentin was attempted, but the reason for its use was rarely described.

### Cytology

We found that almost two-thirds of the UA cytology findings were consistent with C&S results. The most common disagreement was detecting bacteria on cytology that were not subsequently found on C&S, which may lead the veterinarian to prescribe antimicrobials empirically when not required. Many variables influence manual microscopy, including the centrifugation process, slide preparation, and operator interpretation.^19^ Sample storage conditions also can alter microscopy findings. More training in cytology, both in slide preparation and microscopy interpretation could be beneficial for improving the accuracy of in-house UA cytology. This finding also supports the recommendation to perform C&S to confirm the presence of bacteria before starting antimicrobial treatment.

### Subclinical bacteriuria

Antimicrobial treatment is not recommended in patients with subclinical bacteriuria, unless other risk factors are present.^5^^,^^20^ In our study, only 6 of 29 (20%) identified cases of subclinical bacteriuria had appropriate treatment (ie, were not prescribed antimicrobials) before or after receiving C&S results. Concern over the pet owner’s ability to detect urinary clinical signs may lead clinicians to prescribe antimicrobials in these cases. In addition, veterinarians and owners also often are concerned about the health and welfare of the animals, therefore choosing to err on the side of caution and treat these patients.^7^^,^^8^^,^^21^

### Follow-up urine C&S

When sporadic cystitis is treated with antimicrobials and clinical resolution is achieved, current guidelines recommend against C&S after treatment. In the case of recurrent cystitis, veterinarians are discouraged from performing urine C&S during antimicrobial treatment but could consider C&S 5-7 days after completion of antimicrobials.^5^ However, care should be taken when interpreting positive bacterial culture results if clinical cure has been achieved.^5^^,^^13^

Despite guideline recommendations, many veterinarians still perform follow-up UA or C&S regardless of whether clinical signs have resolved. Doing so may reflect a desire to achieve microbiological cure, which is not recommended because subclinical bacteriuria has been found to occur in up to 12% of healthy dogs and cats, with higher rates in animals with comorbidities such as diabetes mellitus, chronic kidney disease, and hyperadrenocorticism.^5^^,^^20^^,^^22^^,^^23^

### Concordance with treatment guidelines

In the intervention group, veterinarians often employed delayed prescribing but uncommonly followed treatment guidelines for prescribing low importance antimicrobials as empirical treatment. Amoxicillin or TMS was prescribed in very few instances in either the intervention or control groups. This finding may be because clinics do not stock these medications, which in part has led to availability issues of these drugs in the appropriate formulation (dosage or administration form), particularly TMS.^24^ Previous studies also found that the “dispensary cycle” (prescribing habits among veterinarians in a practice which influences which antimicrobials are kept in stock in the dispensary) promotes the use of certain drugs over others.^24^

### Limitations

One of the limitations in our study was that the data obtained from clinical records may be inaccurate or incomplete. This possibility is dependent on the pet owner’s observations, the veterinarian’s interpretation of the pet owner’s descriptions, and the completeness of clinical notes recorded by the veterinarian. There also was missing data, such as records of communication with the pet owner and charges for products or services that were not itemized. Clinical records also may be missing if clients visit multiple veterinary clinics. In addition, patient weight was not consistently recorded and thus appropriateness of dosing could not be evaluated.

The control data were limited to the initial presentation for urine testing until 6 weeks after, and thus it was not possible to analyze outcomes related to previous urinary concerns, previous antimicrobial use, comorbidities, and whether or not the urinary clinical signs completely resolved. In addition, full details of C&S results were not accessible in this group.

There may be selection bias for clients who consent to performing UA. If there were financial limitations such that in-house UA is prohibitive, veterinarians may prescribe differently. However, the impact of this factor is estimated to be small because UA frequently is performed, especially in patients with urinary clinical signs, and the cost of UA in Australia is not high.^25^^,^^26^ Previous studies also identified other factors apart from cost that may affect the veterinarian’s decision to perform C&S, such as challenges to obtaining a urine sample and turnaround time for results.^7^^,^^10^

Because all the clinics were located in metropolitan Melbourne, the clinics in our study may not be representative of different types of practices such as clinics located in rural locations or socioeconomically disadvantaged areas, mixed practices, or privately owned clinics. The clinics in this corporate group also have been exposed to AMS interventions previously, and thus some background AMS knowledge is available and there could be a contamination bias if the decision support tool was shared among clinics.

The interventions (free C&S and decision support tool) in our study were assessed as a bundle. It was not possible to distinguish between the effects of the 2 on prescribing behavior. However, previous studies have found implementation of multiple strategies to be more sustainable.^27–29^

### Conclusion

When the barrier of cost was removed and a decision support tool was provided, the frequency of prescribing and duration of antimicrobial treatment decreased. Improvements in clinical record-keeping to include information such as the indication for antimicrobial prescriptions would be helpful for future research in AMS. Further education on diagnostic testing and guideline recommendations also may be beneficial to improve empirical selection of antimicrobials, decrease unnecessary antimicrobial use and increase adherence to guideline-recommended treatment durations.

## Supplementary Material

aalaf054_Supplementary_materials_v2

## Off-label antimicrobial declaration

The authors declare no off-label use of antimicrobials.

## Institutional animal care and use committee or other approval declaration

Approved by the University of Melbourne Human Ethics Advisory Group under the reference number Ref 2022-23395-26121-3.

## Abbreviations

C&S: culture and susceptibility
NSAID: nonsteroidal anti-inflammatory drug
TMS: trimethoprim-sulfamethoxazole
UA: urinalysis
UTI: urinary tract infection
