# Supplementary material for: Antimicrobial prescribing in dogs and cats with urinary tract disease in a prospective intervention trial
Source: J Vet Intern Med. 2026 Jan 21;40(1):aalaf054. doi: 10.1093/jvimsj/aalaf054 (PMC12881951; doi:10.1093/jvimsj/aalaf054)
Supplement: aalaf054_Supplementary_materials_v2 [file aalaf054_supplementary_materials_v2.docx]

SUPPLEMENTARY MATERIALS

Supplementary materials 1

Table SM1: Laboratory culture results from cases of subclinical bacteriuria

| **C&S results** | **Number of samples** | % |
| --- | --- | --- |
| *E. coli* only | 13 | 45% |
| Mixed, involving E*. coli* | 3 | 10% |
| *Enterococcus faecalis* only | 2 | 6.9% |
| *Streptococcus canis* only | 2 | 6.9% |
| Mixed, *E. coli* + *Enterococcus faecalis* | 2 | 6.9% |
| *Proteus mirabilis* | 1 | 3.4% |
| *Citrobacter koseri* | 1 | 3.4% |
| Mixed, involving *Streptococcus canis* | 1 | 3.4% |
| *Pseudomonas aeruginosa* | 1 | 3.4% |
| *Staphylococcus felis* | 1 | 3.4% |
| Mixed, *E. coli* + *Proteus mirabilis* | 1 | 3.4% |
| Mixed, *E. coli* + *Staphylococcus pseudintermedius* | 1 | 3.4% |
| Total | 29 |  |

Supplementary materials 2

Table SM2: Proportion of patients prescribed antimicrobials empirically based on presence of urinary clinical signs and presence of bacteria seen on cytology

| Presence of clinical signs | Cytology performed | Bacteria seen on cytology | Empirical antimicrobials prescribed | Proportion treated empirically |
| --- | --- | --- | --- | --- |
| Yes | Yes | Yes | 59 | 42% |
| Yes | Yes | No | 28 | 20% |
| Yes | No | NA | 26 | 19% |
| No | Yes | No | 10 | 7.1% |
| No | Yes | Yes | 9 | 6.4% |
| No | No | NA | 7 | 5.0% |
| - | unknown |  | 1 | 0.7% |
| Total cases | | | 140 |  |

Supplementary materials 3

Table SM3: Antimicrobials prescribed for empirical and non-empirical (after receiving C&S results) use

| **Antimicrobials** | **Prescribed empirical antimicrobials (%)** | **Antimicrobials only initiated after C&S (%)** |
| --- | --- | --- |
| Amoxicillin-clavulanate | 84 (76) | 23 (66) |
| Cefovecin | 7 (6.3) | 3 (9) |
| Cephalexin | 7 (6.3) | 2 (6) |
| Amoxicillin | 4 (3.6) | 2 (6) |
| Trimethoprim- sulphamethoxazole | 3 (2.7) | 3 (9) |
| Enrofloxacin | 2 (1.8) | 2 (6) |
| Marbofloxacin | 2 (1.8) | 0 (0) |
| Doxycycline | 1 (0.9) | 0 (0) |
| Amoxyclav + Cefovecin | 1 (0.9) | 0 (0) |
| Total | 111 | 35 |
